# Peripheral muscle strength and functional capacity in patients with moderate to severe asthma

**DOI:** 10.1186/2049-6958-10-3

**Published:** 2015-01-21

**Authors:** Elisangela Ramos, Luis Vicente Franco de Oliveira, Audrey Borghi Silva, Ivan Peres Costa, João Carlos Ferrari Corrêa, Dirceu Costa, Vera Lucia Alves, Claudio F Donner, Roberto Stirbulov, Ross Arena, Luciana Malosa Sampaio

**Affiliations:** Rehabilitation Sciences Master’s Program, Nove de Julho University, São Paulo, Brazil; Universidade Federal de São Carlos, São Paulo, Brazil; Santa Casa de Misericórdia São Paulo Hospital, São Paulo, Brazil; Mondo Medico, Multidisciplinary and Rehabilitation Outpatient Clinic, Borgomanero, NO Italy; Department of Physical Therapy, College of Applied Health Sciences, University of Illinois Chicago, Chicago, IL USA

**Keywords:** Asthma, Functional capacity, Obesity, Physical activity

## Abstract

**Background:**

The adequate control of asthma includes the absence of nocturnal symptoms, minimal use of medication, normal or nearly normal lung function and no limitations to physical activity. The choice of a more sedentary lifestyle can lead to physical de-conditioning, thereby aggravating asthma symptoms and increasing the risk of obesity.

**Methods:**

This study aimed at performing a battery of function-related assessments in patients with asthma and comparing them to a healthy control group.

A prospective, transversal and case–control study was designed.

It was set up at Santa Casa de Misericórdia Hospital –Sao Paulo and Nove de Julho University on a population of outpatients.

Subjects of the study were patients affected by moderate to severe asthma.

A case–control study was carried out involving 20 patients with moderate to severe asthma and 15 healthy individuals (control group). All participants underwent body composition analysis (BMI and BIA) and a controlled walk test (Shuttle test), resistance muscle test (1RM) and answered a physical activity questionnaire (IPAQ). The group with asthma also answered a questionnaire addressing the clinical control of the illness (ACQ).

**Results:**

In comparison to the control group (unpaired Student’s t-test), the patients with asthma had a significantly higher BMI (31.09 ± 5.98 vs. 26.68 ± 7.56 kg/m^2^) and percentage of body fat (38.40 ± 6.75 vs. 33.28 ± 8.23%) as well as significantly lower values regarding distance traveled on the walk test (369 ± 110 vs. 494 ± 85 meters) and metabolic equivalents (3.74 ± 0.87 vs. 4.72 ± 0.60). A strong correlation was found between the distance completed and peripheral muscle strength (r: 0.57, p < 0.05) and METs (Metabolic equivalents – minutes/week) and peripheral muscle strength of 1RM (r: 0.61, p = 0.009).

**Conclusions:**

The individuals with asthma had lower functional capacity and levels of physical activity as well as a higher percentage of body fat compared to healthy individuals. This suggests that such patients have a reduced physical performance stemming from a sedentary lifestyle.

Despite the existence of few studies reporting moderate to severe asthmatic patients and functional capacity assessment, it is clear that the assessment presented in the current study is a valid and accessible tool in clinical practice.

## Background

Patients with asthma have diminished activity patterns, leading to deleterious physiologic alterations and ultimately impaired functional capacity. Muscle weakness can be due to reduced motor neuron activity, decreased percentage of type I fibers, increased percentage of type IIb fibers, and reduced activity of enzymes involved in oxidative energy conversion [1, 2]. Patients with respiratory failure, cardiac failure, or both, have decreased peripheral and respiratory muscle strength, although it has been reported that both muscle groups cannot present similar decrease in strength and endurance concurrently [3].

The administration of systemic corticosteroid is a common treatment for asthmatic patients. The effects of corticosteroid therapy on these patients are well known and include the risk for steroid-induced myopathy [4, 5]. Many researchers have reported that acute respiratory myopathy can be caused by therapy with high doses of systemic steroids. However, the chronic consequences to muscle observed in asthma still remain controversial. Respiratory muscle weakness induced by systemic corticosteroids and peripheral muscle alterations have not yet been elucidated in these patients [6–8].

More work is needed to describe and quantify the degree of functional limitations observed in this patient population. Thus, the primary aim of the current investigation was to perform a battery of function-related assessments in patients with asthma and compare them to a healthy control group.

## Methods

### Subjects

This study was conducted on 20 patients with severe asthma and 15 healthy subjects of both sexes. Patients had been under medical outpatient treatment for at least six months prior to participation. They had been clinically stable for three months and were under optimized drug therapy. Inclusion criteria were based on the clinical diagnosis of asthma performed by a licensed pulmonologist. In addition, patients were clinically stable, sedentary, aged 30–50 years, and presented oxygen saturation levels ≥ 92% at rest breathing room air. Exclusion criteria included ischemic heart disease, musculoskeletal disease, and menopause. Subjects in the health control group completed questionnaires and did not present any associated disease. Control subjects were excluded if they were current smokers, taking any type of medication, if they had participated in a regular exercise program in the six months before the beginning of this study, experienced skeletal muscle pain or had difficulty in understanding the exercises of the protocol.

All the procedures were described in detail and all subjects were informed of the experimental risks prior to the study. The volunteers were also informed of the objectives of the study, and written informed consent was obtained prior to study beginning. The investigation was approved by the Ethics Committee for Human Research of Institutions (n. 228471).

### Study design

This is a prospective, transversal, case–control study. The experiments were carried out in a climatically controlled room at 22-24°C and relative air humidity at 50-60%, and performed on different days separated by a seven day interval. Before the day of the experiment, the subjects were taken to the experimental room for familiarization with the procedures and equipment to be used.

The protocol was created on the basis of initial assessment (medical history), body composition [body mass index (BMI) and bioelectrical impedance (BIA)], pulmonary function, incremental shuttle walking test (ISWT), International Physical Activity Questionnaire-short form (IPAQ), and clinical control of asthma.

### Initial assessment

During the initial assessment, information about patient identification, clinical history, associated diseases and medications were collected. This procedure was performed by the physician responsible for the asthma clinic, with the goal of analyzing whether the patient could participate in the research and identifying comorbidities that could hinder physical activity engagement.

### Body mass index and body composition

Body mass index (kg/m^2^) was calculated with the subject barefoot on the Welmy® anthropometric scale, which measures body weight (kg) and body height (m). Body composition assessment was performed by BIA using the digital scale (Tanita Inner Scan®, model BC 554). Prior to testing, patients abstained from physical activity for 12 hours, ingested no alcoholic-beverages for 48 hours, and emptied their bladder 30 minutes before [9].

### Spirometry

Procedures for the pulmonary function test were performed in accordance with the Brazilian Thoracic Society [4] and the American Thoracic Society [6]. The dynamic indices obtained were forced vital capacity (FVC), forced expiratory volume in the first second (FEV_1_) and FEV_1_/FVC ratio.

### Maximum strength (1 RM)

Quadriceps muscle function was assessed by determining the one-repetition maximum (1RM) leg press (Technogym® - model). The 1RM test was applied by gradually increasing the resistance until the subject succeeded in performing no more than one repetition of the exercise on the leg press. Starting position was set at a knee flexion angle of 90 degrees. Subjects attempted successive lifts of single repetition with increasing load until they failed to lift the load through the entire range of motion [10]. The initial resistance load applied to determine 1RM was 80% of the 1RM Endurance (1RM-E), and if the volunteer was able to perform more than one complete movement, the load was increased 10% of the 1RM-E, after the five minutes rest interval between trials. When the first attempt was unsuccessful, because the resistance load had been overestimated, the load was reduced by 10% 1RM-E. When the pre-1RM was determined, a second attempt with an additional 10% above the load was performed to verify the load value and in the cases where the individual was not successful on this second attempt, the previous load was considered as his 1RM; although, if the volunteer was successful, a new load was added until the 1RM was determined. Based on the estimated loads of 1RM, it was expected that 1RM would be determined in up to six attempts [11].

During the test, the volunteer maintained a seated position on the equipment, with knees and hips flexion at 90°. During the movement, the knees and hips were extended and returned to the initial position after the flexion. Before the execution of the test, the volunteer was oriented to avoid the isometric component and exhale during the extension of the knees and hips to avoid the Valsalva maneuver [12].

During the entire test period, peripheral oxygen saturation and heart rate were monitored by a pulse oximeter (Nonin Medical® - model 3100). Blood pressure (BP) was verified by the auscultation method (sphygmomanometer BD, São Paulo, Brazil) before and after each test of maximum load. The noticed level of effort was obtained utilizing the Borg Scale [13].

### Endurance test

Endurance was assessed on a leg press machine (Tecnogym, Italy) with the starting position set at a knee flexion angle of 90 degrees. The endurance exercise test was performed on a separate day; following a one minute warm-up in a low load (10%RM) and after this, the load used was 60% of 1RM with the maximal number of knee extension repetitions carried out to exhaustion [10]. Subjects were encouraged to perform the knee extension as long as possible to determine the exercise tolerability. Time and number of repetitions were recorded. The perception of symptoms of dyspnea and lower limb fatigue were evaluated using Borg’s CR10 scale at the beginning and at the end of the test. Peripheral oxygen saturation (SpO_2_) and heart rate (HR) were also monitored throughout the test. The test finished when the subject could no longer maintain the pre-determined movement cadence and movement angle. The time to failure was described to the nearest second.

### Isometric force

The quadriceps femoris strength was obtained from dominant leg, and the measurement was taken with patients seated on a leg extension at 60° of knee flexion. A non elastic strap connected the ankle to a load cell channel of the acquisition system was enabled for the utilization of the (EMG System do Brazil Ltda), having an output between 0 and 20 mV and a range up to 1 kN. The patients performed three movements of the knee extensors, maintaining each for 5 s, with a minute rest between repetitions.

### Maximal functional capacity

The incremental shuttle walking test (ISWT) was used to assess functional capacity. The ISWT was performed according to Singh et al. [14]. Patients walked back and forth between two cones spaced 10 m apart on a flat surface. Patient’s walking speed was dictated by an audio signal that decreased its interval progressively through the test.

Blood pressure was measured in the left arm with the indirect auscultatory method during the rest period and at the end of the test. HR and SpO_2_ were measured during the test (Nonin Medical® - model 3100). The Borg scale was used to rate the patient’s perceived intensity of dyspnea and lower limb pain at the beginning and end of the test. The intensity of the perceived exertion was graded from zero to ten, with zero corresponding to "no shortness of breath” and ten to “very, very heavy” [13].

The ISWT could be interrupted due to exercise intolerance or terminated by the evaluator. Reasons for test termination included patients’ symptoms (i.e. extreme dyspnea and/or lower limb fatigue) and/or oxygen desaturation. The test could also be terminated when subjects failed to complete the shuttle within the allowed time, i.e. when he or she was more than 0.5 meters away from the cone when the audio signal sounded [14].

Prior to testing, a series of practice trials were administered in order to familiarize the patients with the test procedures.

### Questionnaire on physical activity

The level of physical activity was assessed with the use of the International Physical Activity Questionnaire-short form (IPAQ). This assessment tool was translated and validated in Brazil by Matsudo et al. [15] and its use is indicated for sedentary adult populations (15–69 years of age).

The IPAQ scoring protocol provides categorical and continuous scores. The continuous score expresses the relation between physical activity energy expenditure and time [metabolic equivalents (MET)-minutes/week]. The score is computed by multiplying the energy expenditure value (expressed in METs) of a physical activity (such as walking: 3.3 METs, moderate: 4.0 METs and vigorous: 8.0 METs) by the number of minutes spent in the activity per week. The categorical score classifies individuals as:

Insufficiently active - does not perform any physical activity, or performs physical activity but it is not enough to be classified as moderate or high intensity.

Sufficiently active - performs vigorous physical activity at least three days a week for at least 20 minutes per session; performs moderate physical activity or walk at least five days a week for more than 30 minutes per session; or performs any physical activity with different intensities (walking + moderate + vigorous) more than five days a week and more than 600 MET (minutes per week).

Very active - performs more than three days per week of vigorous physical activity accumulating 1,500 MET; or performs physical activity with different intensities more than seven days a week, covering 3,000 MET [15].

### Statistical analysis

Data are presented as mean ± SD, median, and interquartile after testing for normal distribution (Kolmogorov-Smirnov test). Sample size was calculated using GraphPadStatMate software, version 1.01. Based on pilot study, the number of patients was estimated to be 20 in each group, with a type I error of 5% and a power of 80%. The inter-group differences were assessed by unpaired Student's t test, Mann Whitney test, and Pearson correlation.

A fix was made for lean mass to perform comparisons between the groups, because they show men and women together.

The significance level was set at 5%. Data analysis was performed using the SPSS software package (Version 20.0, Chicago, IL, USA).

## Results

We selected 63 patients with asthma according to the inclusion and exclusion criteria stipulated for the study. Thirty-two patients agreed to participate, 20 attended the assessments while 10 did not. Two patients experienced an asthma attack during the recruiting procedures, thus they were excluded from the study. Baseline characteristics of 20 patients with asthma and 15 volunteers from the control group are presented in Table 1.

**Table 1:** **Anthropometric and demographic data, body composition, spirometric, clinical control of asthma, IPAQ, and medications use**

	Asthma (n = 20)	Control (n = 15)
Sex (M/F)	6/14	1/14
Age (years)	44 ± 6.0	39 ± 6.0
Weight (kg)	79.4 ± 15.5	71.3 ± 18.5
Height (cm)	162.0 ± 8.0	163.7 ± 8.6
BMI (kg/m^2^)	31.1 ± 6.0	26.4 ± 7.6*
PCM (kg)	49.8 ± 9.3	46.8 ± 9.0
Water (%)	44.9 ± 5.2	46.6 ± 5.3
Fat (%)	38.4 ± 6.7	33.3 ± 8.2*
FEV_1_ (% pred)	65 ± 15	102 ± 16.8*
ACQ	1 (0–4,5)	
IPAQ (MET.min) per week		
Inactive	30%	6.66%*
Moderately active	55%	73,33%*
Very active	15%	20%
**Medication, n (%)**		
Inhaled corticosteroids (CI)	12 (60)	-
CI + short-term B-agonists	6 (30)	-
CI + oral corticosteroids	2 (10)	-

Data reported as mean ± standard deviation or median (interquartile intervals). ACQ, asthma control questionnaire; BMI, body mass index; FEV_1_, expiratory volume in the first second; LBW, lean body weight. *p ≤ 0.05.

Peripheral muscle strength determined by 1RM showed no significant differences between patients with asthma and the control group. Table 2 shows the values normalized by lean body mass. The data show a significant difference between the asthmatic and control group.

**Table 2:** **Comparison between patients with asthma and control subjects.**

	Asthma (n = 19)	Control (n = 15)
**Strength exercise test**		
Force isometric (Kgf/Kg)	1.51 ± 0.43	1.74 ± 0.34
Resistance	0.84 ± 0.47	1.41 ± 1.09^*^
METs	0.08 ± 0.02	0.11 ± 0.02^*^
Distance	7.68 ± 2.31	11.40 ± 2.43^*^

Data normalized by lean body mass. *p ≤ 0.05. METs, metabolic equivalent.

Table 3 shows the behavior of physiological variables during the ISWT. We observed significant differences between groups in the distance completed (369 ± 110 vs. 494 ± 85 m), METs (3.74 ± 0.87 vs. 4.72 ± 0.60), end HR (115 ± 15.85 vs. 124.9 ± 21 bpm), and % HR max (65.43 ± 8.88 vs. 69.08 ± 10.44).

**Figure 1:**
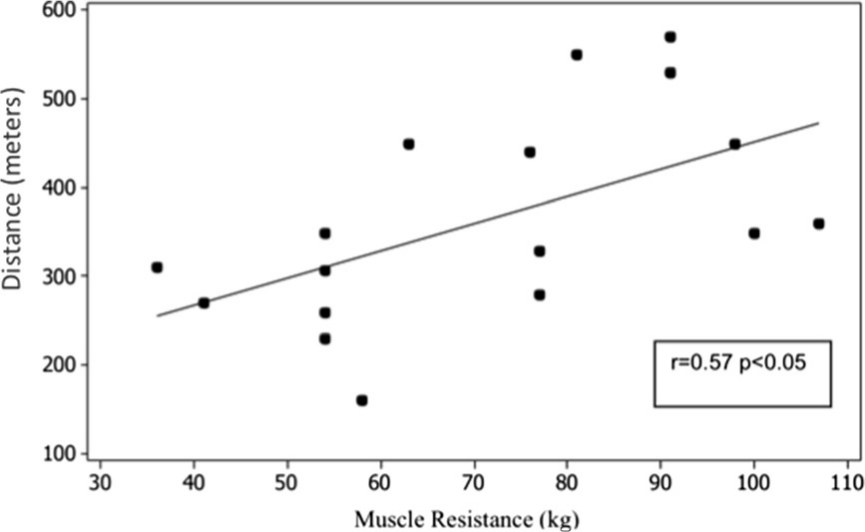
**Distance and muscle resistance.** Muscle resistance (kg).

**Figure 2:**
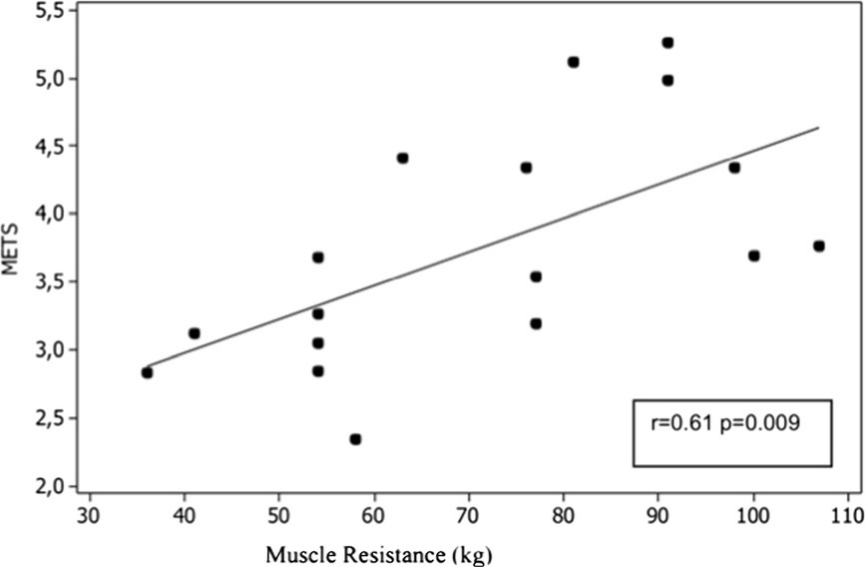
**METs and muscle resistance.**

With respect to the correlations, we observed a significant relationship between the distance completed and peripheral muscle strength gained in the 1RM test (r: 0.57, p <0.05) (Figure 1). Also, a significant correlation was observed between METs and peripheral muscle strength of 1RM (r: 0.61, p = 0.009) (Figure 2).

**Table 3:** **Physical performance and behavior of the cardiovascular and respiratory variables and sensation of dyspnea and fatigue in asthma and control groups**

	Asthma (n = 20)	Control (n = 15)
Distance (m)	369 ± 110	494 ± 85*
METs	3.74 ± 0.87	4.72 ± 0.60*
Initial HR (bpm)	84.14 ± 9.65	85.34 ± 9.64
End HR (bpm)	115 ± 15.85	124.9 ± 21*
Initial PAS (mmHg)	120 (90–140)	110 (90–140)
End PAS (mmHg)	130 (110–160)	120 (100–140)
Initial PAD (mmHg)	80 (60–90)	70 (60–90)
End PAD (mmHg)	80 (60–90)	72 (60–90)
%HR Max	65.43 ± 8.88	69.08 ± 10.44*
InitialSpO_2_ (%)	98 (93–99)	98 (96–99)
End SpO_2_ (%)	97 (84–99)	97 (89–99)
Dyspnea (0–10)	2 (0–8)	1 (0–4)
Leg Fatigue (0–10)	2 (0,5-8)	2 (0–5)

Data presented as mean ± standard deviation or medians and interquartile intervals. %HR max, percentage of maximum heart rate; Borg, sensation of dyspnea; Borg LE, sensation of tiredness and pain in the lower extremity; DAP, diastolic arterial pressure; HR, heart rate; METs, metabolic equivalent; SAP, systemic arterial pressure; SpO_2_, peripheral oxygen saturation. * = p < 0.05.

## Discussion

The main findings of our study suggest that, compared to controls, patients with asthma have reduced functional capacity as characterized by the ISWT and low tolerance during the muscle endurance test. Muscle performance is characterized by muscle strength and endurance. Decreases in any or all of these features result in decreased functional performance [5, 16]. This was observed in our study as a significant difference was detected in muscle endurance between the asthmatic patients and the control group. Several studies assessing skeletal muscle function and functional capacity have been performed in patients with chronic obstructive pulmonary disease. Among the known mechanisms involved with the development of skeletal muscle dysfunction, there are disuse-induced deconditioning, hyperinflation, pro-inflammatory cytokines, hypoxia and/or hypercapnia, malnutrition, and long-term use of corticosteroids [4, 17, 18].

To our knowledge, there are only a few studies addressing skeletal muscle function in patients with moderate to severe asthma [7]. These patients may present pulmonary hyperinflation associated with decreased inspiratory and expiratory muscle strength, which is a critical factor during acute exacerbations of asthma. Patients with asthma may also adopt an inactive lifestyle or decreased physical activity patterns, contributing to obesity and worsening of asthma symptoms. Moreover, they may use corticosteroids during periods of disease exacerbation. We could infer, in the present study, that patients with asthma who use systemic corticosteroids present a decrease in respiratory muscle strength, a premise supported by previous investigations [19, 20].

In addition to the physical inactivity experienced by most patients with asthma, several medications can alter muscle fiber size and type, such as glucocorticoids and β_2_-agonists that are used for respiratory conditions. The use of high doses of glucocorticoids causes atrophy and loss of muscle strength, a condition known as steroid myopathy. This condition impacts the muscle contractile system, which can be severe enough to lead to reduced motor activity and ventilatory performance. In the present study, all asthmatic subjects were under β_2_-agonists and glucocorticoid treatment for at least one year.

Changes in quadriceps muscle strength were not found in the asthmatic patients. Young et al. [18] studied 25 normal subjects (11 men and 14 women) 19 to 48 years old. They found a stronger correlation between cross-sectional area and torque generated by the quadriceps (r = 0.84) than between the last and body weight (r = 0.54). They also reported that there is a decrease in isometric force in elderly females correlated with reduced ultrasound dimension of the quadriceps muscle [18]. Other studies that used computed tomography confirmed a significant positive correlation between quadriceps muscle strength and cross-sectional area [18].

Metabolic processes at the skeletal muscle level due to the use of corticosteroids, whether associated or not with skeletal muscle atrophy, can lead to severe functional impairment. These symptoms are most evident in unusual cases where the onset of such processes is acute. However, the onset of muscle impairment is typically slow and insidious and it is manifested by tiredness, weakness and fatigue during the execution of tasks such as walking or weightlifting.

Further research is needed to better elucidate the assessment of skeletal muscle strength. This will help optimizing endurance training programs for asthmatic patients, which will assist in assuaging the deleterious physiologic and functional impact associated with drug therapy. The assessment of functional capacity of these patients is important for evaluating responses to interventions, prescribing physical training protocols, and determining the level of exercise tolerance [12]. The gold standard in assessing functional capacity is the direct measurement of oxygen consumption (VO_2_) during the cardiopulmonary exercise test. However, it has limitations such as high cost, specialized personnel, and limited availability [12, 18]. These limitations indicate the need for simpler and less costly tests to assess functional capacity. Therefore, this research is of great importance to reduce the costs of assessing functional capacity and monitoring the impact of a rehabilitation program [15, 18, 19].

The ISWT proved to be safe in the assessment of the asthmatic patients’ functional capacity. It was observed in our study that the administration of the test was simple, required simple equipment and could be applied to patients with moderate to severe asthma [15].

The test was easily understood, tolerated, and accepted by patients with asthma. There were no episodes of exercise-induced bronchospasm, dyspnea, or desaturation (below 90%). The distance performed was the main variable observed at the end of the test. The average distance performed was significantly lower in the asthmatic patients compared to controls (369 ± 110 vs. 494 ± 85 m).

Studies have reported that incremental increases in HR and VO_2_ during the ISWT confirmed the physiological response in exercise with the gradual increase in intensity that is similar to the treadmill [15].

The cardiovascular response presented an incremental increase during the test and a significant difference between patients and control group. These findings corroborate the study of Singh et al. [20] where a similar response during this assessment was found.

It was observed that lower limb fatigue, measured with the Borg scale, was higher in the asthmatic group. This response suggests that the exercise limitation presented by patients with respiratory diseases can be triggered by factors such as abnormal exhalation of gases, respiratory muscle weakness, and physical deconditioning [13].

Despite the existence of few studies reporting moderate to severe asthmatic patients and functional capacity assessment, it is clear that the assessment presented in the current study is a valid and accessible tool in clinical practice.

## Conclusion

In conclusion, the results showed a significant difference in skeletal muscle endurance, but not a reduction in muscle strength, when comparing patients with asthma and healthy individuals. In addition, patients with asthma presented greater limitations in functional capacity compared to the healthy subjects. These findings suggest that the loss observed by these patients was probably due to a combination of the disease process, associated pharmacologic management and physical inactivity.

## References

[1] Gosker H, Wouters E, van der Vusse G, Schols AM (2000). Skeletal muscle dysfunction in chronic obstructive pulmonary disease and chronic heart failure: underlying mechanisms and therapy perspectives. Am J Clin Nutr.

[2] Couillard A, Prefaut C (2005). From muscle disuse to myopathy in COPD: potential contribution of oxidative stress. Eur Respir J.

[3] Killian K, Leblanc P, Martin D, Summers E, Jones NL, Campbell EJ (1992). Exercise capacity and ventilatory, circulatory and symptom limitation in patients with chronic airflow limitation. Am Rev Respir Dis.

[4] Cluley S, Cochrane GM (2001). Psychological disorder in asthma is associated with poor control and poor adherence to inhaled steroids. Respir Med.

[5] Fahy JA, Kim K, Liu J, Boushey HA (1995). Prominent neutrophilic inflammation in sputum from subjects with asthma exacerbation. J Allergy Clin Immunol.

[6] Fahy JA, Liu J, Wong H, Boushey HA (1993). Cellular and biochemical analysis of induced sputum from asthmatic and from healthy subjects. Am Rev Respir Dis.

[7] Mckenzie DR, Gandevia SC (1986). Strength and endurance of inspiratory, expiratory, and limb muscles in asthma. Am Rev Respir Dis.

[8] Ram FS, Robinson SM, Black PN, Picot J (2005). Physical training for asthma. Cochane Database Syst Rev.

[9] Sociedade Brasileira De Pneumologia E Tisiologia (2006). IV Brazilian Guidelines for the management of asthma. J Bras Pneumol.

[10] Brown HM (1958). Treatment of chronic asthma with prednisolone; significance of eosinophils in sputum. Lancet.

[11] Decramer M, Stas KJ (1992). Corticosteroid-induced myopathy involving respiratory muscles in patients with chronic obstructive pulmonary disease or asthma. Am Rev Respir Dis.

[12] Padula CA, Yeaw E (2007). Inspiratory muscle training: integrative review of use in conditions other than COPD. Res Theory Nurs Pract.

[13] American College Of Sports Medicine (2003). Acms’s guidelines for exercise testing and prescription.

[14] Weiner P, Berar-Yanay N, Davidovich A, Magadle R, Weiner M (2000). Specific inspiratory muscle training in patients with mild asthma with high consumption of inhaled beta(2)-agonists. Chest.

[15] Baldwin KM, Haddad F (2002). Skeletal muscle plasticity: cellular and molecular reponses to altered physical activity paradigms. Am J Phys Med Rehabil.

[16] Rizzo MC, Sole D (2006). Inhaled corticosteroids in the treatment of respiratory allergy: safety vs efficacy. J Pediatr.

[17] Young A, Stokes M, Crowe M (1985). The size and strength of the quadriceps muscles of old and young men.. Clin Physiol.

[18] Young A, Stokes M, Crowe M (1984). Size and strength of the quadriceps muscles of old and young women. Eur J Clin Invest.

[19] Win T, Jackson A, Groves AM, Sharples LD, Charman SC, Laroche CM (2006). Comparison of shuttle walk test with measured peak oxygen consumption in patients with operable lung cancer. Thorax.

[20] Singh S, Morgan MD, Scott S, Walters D, Hardman AE (1992). Development of a shuttle walking test of disability in patients with chronic airways obstruction. Thorax.

